# Perceived health status in a comparative perspective: Methodological limitations and policy implications for Israel

**DOI:** 10.1186/s13584-016-0128-x

**Published:** 2017-01-13

**Authors:** Baruch Levi

**Affiliations:** The Israeli Medical Association (IMA), 35 Jabotinsky ST., Ramat Gan, 52136 Israel

**Keywords:** Perceived health status, OECD, Better life index, Health measurement, Health policy, Israel

## Abstract

**Background:**

The perceived health status indicator included in the OECD Health Statistics suffers from severe methodological limitations related to data collection. Furthermore, this indicator is also included in the OECD's Better Life Index, thus distorting the total health score of some OECD countries, among them Israel. The purpose of this paper is to explore the erroneous use of OECD health data in Israel and to warn of its implications.

**Methods:**

Analysis of data from the OECD Health Database, Better Life Index and the Israeli Central Bureau of Statistics, review of media reports and governmental documents concerning health measures, conversations and correspondence held with officials in the relevant organizations.

**Results:**

OECD's perceived health status outcomes for Israel are biased upwards, resulting also in an upward bias of the Israeli overall health grade in the Better Life Index. This is due to the methodological differences between the OECD's standard survey questionnaire and the Israeli one. Yet, erroneous comparisons constantly appear in governmental documents and media reports, presenting health status in Israel in an excessive positive light.

**Conclusions:**

Data from the OECD Health Statistics and the Better Life Index are reaching policy makers and the public in a manner that potentially distorts professional and political discourse on health. This may lead to a decrease in the resources allocated to health based on a flawed comparison. In the long run, and no less serious, the systematic imprecision may detract from the reliability of authority reports in the eyes of the public. Caution is essential in dealing with health indices and international comparisons. The OECD and relevant national agencies should invest greater efforts in the consolidation of definitions and methodologies.

## Background

In recent years, governments and national agencies are investing great effort in measuring the performance and outcomes of public systems [1, 2]. This trend is compatible with the attempts by various entities around the world to develop new measures of quality of life as an alternative, or as a supplement, to the traditional measurement of financial indicators such as Gross Domestic Product (GDP) and income per capita [3, 4]. At the same time, the health sector particularly uses multiple indicators that measure various aspects of health within the population and health systems. International comparisons and various rankings of countries and health systems are published from time to time by parties including the World Health Organization (WHO) and the Organization for Economic Cooperation and Development (OECD) [5, 6]. Databases maintained by these organizations contain dozens of indicators that present various quantitative aspects of health including life expectancy, infant mortality, mortality and morbidity rates, along with health system infrastructure data including number of hospital beds, physicians and nurses and their ratio compared to the population and various process and outcome measures of quality in medical care.

One of these indicators that is of particular importance when it comes to measuring the health of populations is "perceived health status". This indicator is based mainly on survey questionnaires that contain, in various formulations, the question: How do you assess your health? The possible answers to this question lie in a number of choices between the best and worst situation. The response is supposed to reflect the subject's feeling regarding the state of his health [7].

In the annual data published by OECD about the state of health in the 35 member countries, Israel is constantly prominent in the high percentage of citizens who define their condition as "very good" or "good".^1^ This percentage is one of the highest among organization members and is generally on the rise. On this indicator, and in other health indicators including life expectancy and infant mortality, Israel's outcomes are among the highest worldwide [5, 6].

Furthermore, the OECD publishes its unique annual quality of life index (Better Life Index - BLI). This index is composed of several categories of measures whose content attests to the quality of life in the country, e.g. education, environment, employment and housing. One of these categories is health. The overall health score is comprised of the scores on two equally important indicators on which Israel particularly excels – life expectancy and perceived health status. Therefore, Israel is among the leaders on this category among the world's economically developed countries [8].

At face value, it looks like a fairly positive reflection of Israeli society. However, more detailed study of the measurement methods reveals severe methodological limitations preventing acceptable international comparison of some OECD countries, including Israel.

In this article, I will present the methodological problems involved in international comparison of self-assessment of health published by OECD, with a particular reference to Israel. I will then show how this comparative data is placed before policy makers, the media and the public and point out the hazards involved in using this data and publishing it when formulating health policy on a national level.

## Methods

The analysis of the methodologies used to collect and process the data for OECD’s “perceived health status” indicator was done in the following way: Several databases maintained by the OECD and the Israeli Central Bureau of Statistics (CBS), namely the OECD Health Statistics, the OECD BLI and the CBS social survey, were explored. A comparison between the formats of the survey questionnaires was performed, followed by a comparison of the surveys' findings in each OECD country, with an emphasis on Israel.

Later on the investigator reviewed press reports and official reports by government bodies published in Israel between 2011 and 2016 to assess the presentation of the “perceived health status” indicator. Based on these publications the investigator identified hazards deriving from reliance on international comparisons with a meager methodological base in policymaking and on the media.

Less formally, the investigator also had several interactions (conversations and correspondence) with official parties in the OECD and the CBS. These interactions helped him comprehend these parties' approach to the data underlying the "perceived health status" indicator and the methodology on which it is based. They will be discussed further below.

## Results

Every year, the OECD publishes a collection of more than 1,200 indicators that assess the state of health and the health systems of its member countries. These indicators are divided into several content categories including health status, health care resources, health care quality indicators etc. On each category, comparison may be made between member countries on an annual basis.

Clearly, the reliability of the comparison primarily depends on the uniform definition of the measured indicator and method of measurement in each country; otherwise, the international comparison becomes invalid. In indicators based, for example, on surveys held among sample populations in different countries, the use of a survey questionnaire phrased in the most uniform way possible is of utmost importance. However, this condition does not exist in the case of the "perceived health status" indicator. The methodological variation among survey questionnaires influences the results of the international comparison and that in its turn influences the above-mentioned BLI, published by the OECD in recent years and which has been gaining a lot of public attention.

The OECD defines perceived health status in the following manner [7]:

"*Percentage of the population, aged 15 years old and over who report their health to be ‘good/very good' (or excellent) (all positive response categories), ‘fair’ (not good, not bad), ‘bad/very bad’ (all negative response categories)*".

Most countries, mainly European countries, use a joint survey questionnaire in which the question "how is your health in general?" may be answered with one of the five following choices: very good, good, fair, bad, very bad.^2^ As mentioned in the above definition, the OECD divides the results into three categories that it publishes in its database:

- **A positive health assessment category** comprised of the proportion of individuals that selected one of the two positive choices (very good or good),
- **A neutral category** containing the proportion of individuals that chose the middle choice (fair) and
- **A negative health assessment category** that includes the proportion of those who chose one of the two negative choices (bad or very bad).

In technical documents that accompany the database, the OECD notes it has still not achieved full uniformity of this indicator among member countries and indeed, several countries use differently phrased questionnaires: Australia, Canada, Chile, Israel, New Zealand and the USA [7].^3^

In what way do these countries differ from the rest of the OECD and how are their outcomes influenced by the survey questionnaires?

In order to answer these questions, one must separate Israel from the rest of the group inasmuch as Israel is a unique case not only compared to the deviating countries but also compared to all other member countries – and therefore constitutes a particularly problematic case methodologically speaking.

Australia, Canada, Chile, New Zealand and the USA use a common formulation. As the responses to the question about the state of their population health (phrased almost identically), they use the five following choices: excellent, very good, good, fair or poor (in Chile: "regular" and "bad" instead of "fair" and "poor"). In these countries, too, the OECD uses the same principle of grouping the responses into three categories (positive, neutral and negative), only in their case, **three** of the options are combined into a positive category (excellent, very good, good), and there is only **one** option (poor) in the negative category.

**Fig. 1:**
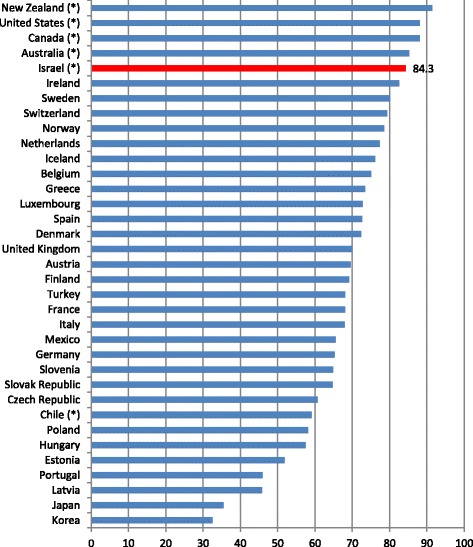
Perceived status of health: % positive responses (OECD 2016). (*) Countries whose survey questionnaire differs from the OECD uniform questionnaire. Data from most countries relates to 2014

In contrast, the standard survey questionnaire includes **two** positive and **two** negative options. Hence, the aggregate percentage of all subjects that select responses in the positive category in these countries is upwards biased and this gives them an advantage over the countries that use the standard survey question. Figure 1 illustrates this.

The perceived health status indicator data in Israel is based on the social survey carried out by the CBS annually since 2002. The social survey is composed of two major sections: the regular core that contains a large number of questions on various aspects of living including health, housing, employment, education, financial situation, use of computers, religion and religious practice etc., and a rotating section dedicated to one or two new subjects every year, studied extensively [9].

The perceived health status question is part of the regular core. Its findings are sent by the CBS to the OECD in the fashion used by other member countries in which different entities are responsible for collecting and processing statistical data and transferring their information to the organization. However, unlike other countries, in response to the question "what is your physical condition in general?" the Israeli questionnaire provides the subject with only four choices instead of five, without a neutral choice, i.e. all the choices are either positive or negative (very good, good, not so good, not good at all) [10].^4^ Israel is the only OECD country that takes this approach. Without the option for a neutral response, all subjects who respond to the perceived health status question must provide responses that fall in either the positive (i.e. very good or good) or the negative category (not so good or not good at all). Thus, Israeli data in the OECD database on the percentage of the population with positive perceived health status is biased upwards compared to countries that use the standard questionnaire.

**Fig. 2:**
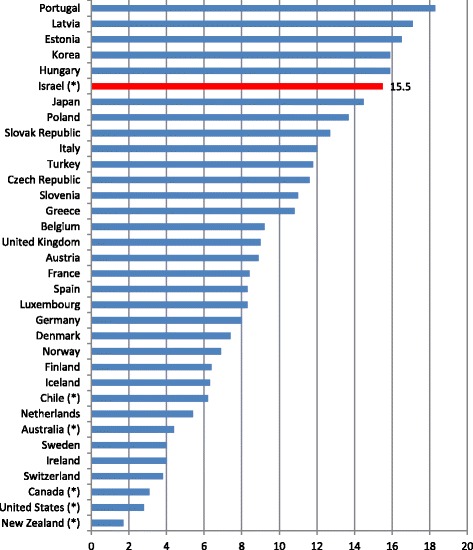
Perceived status of health: % negative responses (OECD 2016). (*) Countries whose survey questionnaire differs from the OECD uniform questionnaire. Data from most countries relates to 2014. Mexico was not included in the comparison due to missing data. For an unclear reason, this country only sends data from the positive category to the OECD

Figure 1 shows that five of the six countries using the differently phrased questionnaire are in the five top places on OECD (except Chile that lags behind the five other countries on important financial measures including GDP per capita and national expenditure on health). Israel is located in the fifth place with more than 84% of its subjects providing positive responses. However, due to the particular formulation of the Israeli questionnaire, the Israeli data on the percentage of the population with negative perceived health status is also biased upwards. Figure 2 provides indication of this.

Israel is among the leading countries on both positive perceived health and negative perceived health. On the other hand, the four other countries leading on the positive category, that also have questionnaires that deviate from the standard, are at the bottom of the negative category (i.e. a very small percentage of subjects perceive their physical condition as negative). As mentioned, unlike Israel, these countries provide the subjects with a neutral choice and only one negative choice instead of two.

Israel is the only OECD member country at the top of both positive and negative rating (among the ten leading countries). This anomaly is related to Israel's unique methodology that deviates from the standard accepted by the organization, raises questions regarding to the state of health in Israel: Is it so good? On the other hand, is it so bad? The answer to this question at the moment seems to be: depends on how the picture is presented.

The problem is further stressed in view of the publication of the OECD BLI, since on this index the "perceived health status" indicator has a key position. The popularity of the index among policy makers and the media turns academic-methodological deliberation of the question of the measurement of this indicator into a political and public issue that should not be ignored. It should place a warning signal before decision makers, health organization, the media, the public at large and other interested parties with regard to the "treatment" and interpretation they give to health indices.

### Perceived health status, state of health and OECD Better Life Index

BLI is an index launched by the OECD in 2011, and since then published annually as a part of the organization's continuing effort to measure well-being and societal progress (the Better Life Initiative). The index is a pioneering attempt to present in a comparative way various aspects of quality of living in different countries, following the general agreement among experts that simple financial measures such as GDP per capita, cannot attest to quality of life. The index includes 11 different categories relating to living: housing, income, jobs, community, education, environment, civic engagement, health, life satisfaction, safety and work-life balance [3]. Each category is composed of the combination of one to four specific indicators presenting different aspects of its content world. The score awarded to each country on each of the indicators is derived from its position compared to the other countries. Thus for example, the country with the highest life expectancy will receive the score of 1 and the one with the lowest life expectancy will receive zero. The remaining countries will be scored according to their position on this continuum [8].

The scoring formula is:

$$ \frac{\mathrm{Value}\ \mathrm{t}\mathrm{o}\ \mathrm{convert}\hbox{-} \mathrm{minimum}\ \mathrm{value}}{\mathrm{Maximum}\ \mathrm{value}\hbox{-} \mathrm{minimum}\ \mathrm{value}} $$

When the indicator is negative (e.g. "rate of murder cases" in the "safety" category) the formula will be:

$$ 1\hbox{-} \frac{\mathrm{Value}\ \mathrm{t}\mathrm{o}\ \mathrm{convert}\hbox{-} \mathrm{minimum}\ \mathrm{value}}{\mathrm{Maximum}\ \mathrm{value}\hbox{-} \mathrm{minimum}\ \mathrm{value}} $$

Each indicator has equal weight. Therefore, the final score on each area is the mean score of the indicators converted into a scale between zero and ten.

The health category is comprised of the two following indicators: life expectancy and perceived health status. The OECD determined that the perceived health status indicator should be the aggregate proportion of subjects on the positive assessment category. Thus, the score awarded to Israel may be calculated in the following manner^5^:

Life expectancy from birth (years):

- Maximum = 83.4 (Japan)
- Minimum = 56.8 (South Africa)^6^
- Israel = 82.1

Normalized score for Israel according to the above formula: 0.951

Perceived health status (percent):

- Maximum = 90 (New Zealand)
- Minimum = 35 (South Korea)
- Israel = 80

Normalized score for Israel according to the above formula: 0.818

The general health score (after averaging the two normalized scores and converting to a scale between zero and ten): 8.85 (or rounded to 8.9 on the BLI website) which puts Israel in the seventh place among the 38 countries.

At this stage, the question arises as to how Israel's position would change had we modified the measurement method somewhat and exchanged the positive health assessment category for a negative one (i.e., instead of using the aggregate percentage of individuals that perceive their health as good or very good, we would have used the percentage of those that perceive it as bad/very bad. Naturally, the smaller their number, the better the general health condition). This form of measurement equals the present form in its methodological and theoretical legitimacy. Moreover, it can be argued that from a normative aspect it even exceeds it: A society could, and perhaps should, be assessed by its approach to its weaker members and definitely those that require medical assistance above all. Therefore, from a social viewpoint, when examining questions relating to the quality of living of human societies it is better to focus on the percentage of those that suffer the most instead of those whose personal situation, according to their testimony, is good to excellent.

I will now recalculate the perceived health status index according to the negative health category for the 34 OECD nations^7^:

Perceived health status (percent):

- Maximum = 20 (Israel)
- Minimum = 2.3 (New Zealand)
- Israel = 20

Normalized score: 0

One may observe that because the Israeli data is the maximum point (and therefore the most negative in this case); Israel's indicator score is zero. Therefore, Israel's overall health score according to this calculation is 4.75. Israel's position thus falls down to the 31st place at the bottom of the list, preceding only Estonia, Hungary and Latvia.

Of course, as mentioned earlier, the omission of a neutral choice from the Israeli survey questionnaire "blows up" the aggregate percentage of subjects in the negative health perception category. However, had the OECD decided to use it instead of the positive category, Israel's condition compared to the other countries would have been the opposite of its current position. The picture would have been turned upside-down without changing a thing on the selected indicators, their weight and the assessment methodology. Nothing has changed in the data collected in the Israeli survey regarding perceived health status. They were not re-calculated or subject to any extrapolations. The only change was in selection of the assessed category from within the indicator, which at least according to the author is preferred to the existing situation from a social viewpoint.

One may assume that had Israeli data been based on the standard survey, Israel's location on the health category would have been somewhere between its present location and its position based on the calculation suggested above, since a neutral option would have absorbed some of the subjects both from the positive and negative categories. Therefore, Israel's final result would probably have been more moderate.^8^ One must also note Israel's perceived health status is on a continuous rise. On the other hand, that is the case in many other OECD states, and since BLI is a relative index, it is hard to say whether Israel's position would necessarily improve in future.

### Careful, indices ahead! Between measurement and policy

The OECD does note in its publications the methodological variance between countries and warns of comparing them; however, these clarifications are sometimes "hidden" in the small letters and in database technical-methodological notes [3, 5]. The actual index receives massive media coverage, while the methodological notes receive virtually no media attention. The health score attracts particular attention, being Israel's highest score from among the different aspects, while its score on other areas is mostly medium or less. Israel's health situation gains many compliments and the score it receives is presented as an impressive achievement, along with attempts to explain it usually through the extraordinary efficiency of the health system or a particularly healthy lifestyle.

This for example, is how "The Marker" newspaper reported on the Better Life Initiative [11]: "…*Despite the small investment in the health system, the percentage of those reporting good physical condition in Israel is higher than in other member nations. The writers of the report noted the data is impressive considering the fact that national expenditure on health in Israel (both per capita and as a percentage of GDP) is lower than the average in OECD and Israel's health system is particularly efficient. The organization notes that perhaps Israelis' health is better due to a healthier lifestyle – they consume less sugar and alcohol and more fruits and vegetables compared to other members, and the percentage of smokers is lower*".

One may wonder how these explanations, true or false, would be compatible with the alternative outcome calculated above or alternatively how those parties would have explained a relatively moderate outcome that would probably have been produced had Israel used the standard questionnaire recommended by OECD.

The Haaretz newspaper also discussed Israel's high position in the health category. The surprising Israeli outcomes were called in an article "the Israeli paradox", i.e. excellent health outcomes gained with relatively low investment in health services infrastructure, despite claims made by physicians and other professionals that the health system is in dire straits. A series of experts interviewed for the article offered explanations for the paradox seemingly deriving from BLI findings [12]. Needless to say, a series of objective health and medical indicators (e.g. infant mortality and life expectancy) indeed hint at the existence of this type of paradox. However, if it does or does not exist, surely nothing may be concluded about its existence from the BLI index as long as it is calculated in its present form. In any case, in its present form, the index supports the contention that a health paradox indeed exists and thus supports parties wishing to curtail the costs of the health system. As the reporter aptly described in his article [12]: "…*This data joins messages that Ministry of Finance (MoF) Budget Division officials have wished to convey in recent years: against the background of physicians' and nurses' strikes, the MoF consistently pointed out that Israel's position is at the top of the rating on international comparisons of leading health indicators, e.g. life expectancy, infant mortality and fertility rate*."

Indeed, in its health budget proposal for 2017–2018 the MoF argued for the "*proven success of the public healthcare system in preserving and improving the population's health*" and for the "*satisfaction of the Israeli population of the healthcare system*". The MoF supported its contentions with a series of international comparisons, highlighting Israel's impressive outcomes in several important health measures such as life expectancy and Infant mortality. It also presented the high proportion of Israelis perceiving their health as good or very good in comparison to other OECD countries, without mentioning the methodological limitations of the comparison that inflates the Israeli outcome [13].

In 2012, the MoF made an international comparison based on OECD data. The document itself was not published, but its data was provided to the Globes newspaper [14]. The article praised the state of health in Israel and among other things said "*Israelis are extremely satisfied with the quality of their health*". The MoF research and economics division was quoted in the article, saying "*Israel excels in public health*". In correspondence with the MoF I was provided with the OECD BLI data quoted in the article. These documents served as a basis for the erroneous comparison [15]. In its series of "How's life" publications dating from 2011, the OECD "red-flags" countries whose perceived health status data is incomparable due to methodological differences, but only in the 2015 publication Israel was finally added to this group [3, 16, 17].

The Bank of Israel's report from 2012 also quoted BLI findings. The report noted Israel is above average in health as well as several other aspects. In this part of the report, the Bank of Israel found a connection between GDP per capita and economic welfare, therefore the Israeli case regarding health was again perceived as unusual: a country with relatively modest financial means (compared to most economically developed countries) that manages to produce excellent health for its citizens [18]. Here too, the message that arises from this (even though not stated explicitly) is that additional investment in health may be unnecessary, based on the BLI index.

BLI findings made their way to "the Caesarea Forum", an annual economic convention held by the Israel Democracy Institute. There the index received criticism in a discussion of inequality in health [19]. However, the criticism did not relate to the methodological distortion discussed in this paper; instead, it focused on the fact the average measured by the index conceals wide inequalities in health that are not expressed – a true and justified claim in itself, but also true when it comes to many other indicators. As for the index findings, it was claimed, again, that Israel was in a high position.

What about the Ministry of Health (MoH)? The MoH also takes pride in Israel's achievements relating to health [20]. It notes on its website that "***the Israeli health system is located in extremely high positions with regard to efficiency of the health system and its results*** (original bold letters). *On the organization's quality of life Index, Israel is seventh when it comes to health and on the efficiency index that examines the potential gain in life expectancy Israel is ninth among women and seventh among men*."

The Ministry links the BLI index outcomes to another form of assessment that focuses on the efficiency of the health system in terms of life expectancy. It thus supports the assumptions of its predecessors regarding the efficiency of the health system, again without relating to biases in the index that do not allow acceptable comparison between Israel and other countries.

## Discussion

**Fig. 3:**
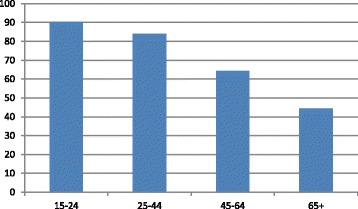
Perceived status of health: % good/very good responses by age group – average of OECD member countries (2014)

Except for the use of questionnaires that differ from the methodology instructed by the organization, the most prominent common denominator among the deviating countries is their population that is on average slightly younger compared to the population of other economically developed countries, e.g. Western Europe, Northern Europe or Japan. Arguably, younger populations tends to have better health and therefore – tend to have higher personal assessment of their health, as evident in the following figure which indicates that the proportion of positive reporting of health status decreases with age [5] (Fig. 3).

**Fig. 4:**
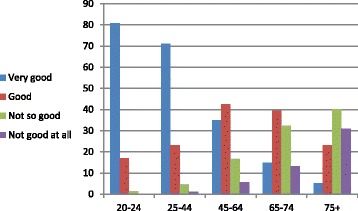
Perceived status of health: Israeli data by age groups (2013)

Figure 4 further supports the argument that younger populations report better health by examining the Israeli CBS data when the population is divided into five age groups. Again, positive reported health status decreases with age and negative reporting increases [9].

The age impact is particularly important in the Israeli case in which the percentage of 65-plus is 11% of the population while in countries such as Germany, Italy and France it is 20% or more [21]. Since OECD data are not standardized for age, one may assume this variable also contributes to Israel's place at the top of the ranking [22]. On the other hand, there are several other countries such as Iceland, Luxemburg and Norway whose percentage of elderly population are relatively small as well and are not ranked at the top five. These countries use the regular OECD questionnaire.

However, it is difficult to point out additional factors that could influence self-assessment of health common to all five leading countries and setting them apart from all the others. In fact, the relevant economic and structural differences between these countries are quite apparent. They are expressed in the type of health systems they have (public compared to private funding, e.g. New Zealand versus USA), physical condition of the population (e.g. great difference in life expectancy and infant mortality between Israel and the USA) and their financial situation (different levels – with great difference between countries – of GDP per capita) [5]. Seemingly, the prominent economic property shared by all – a relatively high level of economic inequality compared to other developed countries – should have negative influence over their outcomes and somewhat balance the influence of the younger population, assuming economic inequality has negative influence over the health of the entire population [23–25].

Perhaps, in addition to all the above, there may also be cultural differences that could influence the results. Inter-cultural differences expressed in surveys have been reported in the literature. It was thus found that Hispanics tend to select responses that are more extreme than those chosen by white Americans on the five-option scale [26]. OECD reports a cultural tendency among the Japanese to give more moderate answers, less radical or "extroverted" compared to citizens of other countries (central tendency bias) [27]. However, standardizing results by cultural tendencies faces great methodological and theoretical obstacles. Furthermore, it is difficult to define cultural variables, assess their influence or point out the causal mechanism through which they operate. In any case, according to the OECD, despite the subjective nature of this question, it turns out the responses manage to provide a good forecast of the future use the person will make of health services [8]. Thus the great importance given by the OECD as a variable that attests to the state of the population's health.

In summary, taking into account the different variables that could influences the "perceived health status" indicator; it seems that the outcomes were heavily influenced by the methodological deviation in the questionnaires, thus contributing to the high position of the five leading countries. My correspondence with OECD officials indicates the organization is aware of the problem of positive deviation common to these countries and agrees with this assessment. The organization further stated it was only able to encourage national authorities to use a uniform questionnaire but is unable to force it on them [22].

The findings indicate that the influence of the BLI index is reaching policy makers as well as the public through the media and messages issued by public officials. These reports do not attend to the methodological problems involved in measurement and do not mention that these problems severely compromise the ability to compare Israeli data to that of other nations. The excessive positive light on Israeli data generates a mistaken professional and public discourse that may give rise to unwanted results. The major risk is that decision makers and the public will be convinced by imprecise measurement and irresponsible publications that the state of health is better than it really is. As a result, decisions may be made to channel societal resources to other areas in which Israel ranks much lower than in health. Distorted reading of the outcomes may cause distortion in allocation of resources in a way that may be hard to set straight subsequently.

This issue has recently become particularly important as the Israeli government declared its commitment to the development of quality of life measures and their integration into socio-economic policy processes in two government decisions from 2012 and 2015 [28, 29]. Health measures are included in this initiative, with perceived health status among them. But even in a report that discusses the initiative and was presented in 2015 in a meeting of the Public Advisory Council for Statistics, which among its duties advises the government in statistical issues concerning state affairs, there seems be no reference to the methodological limitations of the international comparison of perceived health status. Israel is once again presented as one of the OECD leaders of this particular indicator [30].

The fact that this is not an anecdote or a single case that does not attest to the overall situation is no less problematic. Recently, the Taub Center published a study about waiting times for medical services in Israel, including waiting times for elective surgeries, based on OECD data. Again, the comparative view compliments Israel, but the researchers noted the differences in the measurement methods, including the question – what defines a "queue" (how is its starting point determined). They noted that Israeli data might be distorted downwards, i.e. – queues in Israel may be longer than published compared to other countries [31].

Another indicator of great importance that received harsh methodological criticism in recent years is the number of physicians in Israel compared to the size of the population. Reports published by the Knesset Research and Information Center and the Israeli Medical Association (IMA) explained in detail why the ratio of physicians in Israel is probably lower than that published on the organization's database [32, 33]. It is mainly related to different ways to count physicians (those licensed to practice medicine compared to the number of physicians actually practice medicine) and to the lack of consistent, accessible and updated information on the number of physicians active in Israel due to the lack of periodic licensing and registration mechanisms employed in other countries.

One should note carefully that in all the above cases the methodological differences divert the data in favor of Israel, not the other way around. Naturally, national authorities find it more convenient to accept methodical imprecision in publications when they flatter the nation's overall situation, even if it is not carried out by them on purpose. One may assume that government agencies and ministries are happy to present the public and decision makers with data that shed positive light on the areas under their control. However, they must consider the implications that may arise. First of all, such assessments may, as mentioned above, distort government policy, mainly with regard to allocation of resources. Another risk is that insufficient effort will be put into addressing important (but little known) problems. In the long run, and no less serious, the systematic imprecision may detract from the reliability of authority reports in the eyes of the public, thus eroding its faith in government institutions. This point is particularly important in the case at hand in view of the fact the social survey was held by CBS ordered by no other than the MoF budget division, the party that controls allocation of public resources, including the health budget.

It was clear from correspondence held with OECD officials that they are familiar with the Israeli exceptionality and therefore call for a cautious approach in handling OECD (and particularly BLI) data [22, 34]. CBS officials are also well aware of the current situation [35, 36]. But in the case of the CBS, the change in measurement methodology involves "breaking the series" of existing data since 2002. Arguably, no professional entity that deals with measurement takes this step easily. On the other hand, isn't this a price worth paying for the benefit of a proper international comparison?

Israel's joining the OECD in 2010 was celebrated with great joy. Hasn't the time come to make the changes required to respond to the organizations demands and procedures? One must keep in mind that the organization invests great efforts in collecting data from member countries to enable viable international comparisons that support research on these issues and aid the formulation of health policy.

## Conclusions

Systematic measurement of various aspects of living is required for assessment of populations' quality of life. This measurement, carried out by OECD through BLI, is innovative and arouses great public interest. BLI is composed of a large number of indicators, some taken from the organization's health database. Despite the great effort invested in measurement and despite its getting more sophisticated, consolidation of definitions and methodologies for the collection and processing of data has yet to be completed. This is prominent in the data of the "perceived health status" indicator published by the organization, particularly with regard to Israel. The OECD uses data about perceived health status provided by the CBS. Due to the unique character of the questionnaire used by CBS compared to the standard survey used in most member countries, the Israeli outcomes on this indicator tend to be extreme – both positively and negatively.

The lack of methodological uniformity does not allow viable international comparison. The aggregate proportion of those that perceive their health is positive constitutes half the general health score of each country in the BLI. The Israeli score, upwards biased, places Israel at the top of the ranking, while use of the negative category would have positioned it at rock bottom.

The OECD is aware of this problem and therefore publishes warnings regarding the use of its comparisons, but these warnings are not expressed in media coverage and Israeli government reports. Due to the remarkable attention the index receives, it seems to enhance the impression among decision makers as to Israel's excellent health results compared to its relatively small investment in its health system, although correct comparison would probably have yielded a more modest result. This state of mind among decision makers and the public may contribute to a decrease in the resources allocated to health based on an unacceptable measurement. Other examples mentioned in relation to other indicators, show it is not an isolated case.

Great caution is needed when dealing with indices and international comparisons regarding health, due to their complexity and the methodological difficulties they involve. The authorities trusted with measurement in Israel and in the OECD should invest greater efforts in making the definitions and methodologies on which international comparisons are based more uniform. In addition to the risk of distorted resource allocation, publication of unacceptable comparison may erode public trust in government institutions.

## Abbreviations

BLI: Better life index
CBS: Central bureau of statistics
ESS: European social survey
GDP: Gross domestic product
IMA: Israeli medical association
MoF: Ministry of finance
MoH: Ministry of health
OECD: Organisation for Economic Cooperation and Development
WHO: World Health Organization

## Footnotes

1 In addition, OECD also publishes perceived health status from Lithuania that is not an OECD member.

2 There could be slight variations in the phrasing of options as in the cases of Japan and Mexico (for instance "regular" instead of "fair").

3 Differences may also be found in the minimum age of the sample population, however they are relatively negligible. In some of the countries, subjects are 15 and over, and in others the minimum age is 16. In Israel, the survey is administered to people over 20.

4 Alternatively, the subject may say his condition is unclear to him or refuse to answer.

5 BLI 2016 is based mainly on data from 2013.

6 The index also includes three non-OECD countries: Brazil, South Africa and Russia.

7 This calculation is based on OECD health data for 2013, which unlike BLI, do not include Brazil, South Africa and Russia, non-OECD members. Mexico is a member, but for an unclear reason it only sends the OECD the positive category data and therefore is also not included in the alternative rating I created. Hence my calculation includes 34 countries instead of 38.

8 Although caution should be exercised when comparing different surveys with different methodologies, indication of that can be found in the European Social Survey (ESS) which includes Israel. ESS is an academically driven cross-national survey that has been conducted across Europe since 2001 every two years. The survey includes a question of subjective general health with the same five-option scale recommended by the OECD. Israel's aggregated proportion of positive responses (good / very good) in this survey reached 74% in 2012 and 72.4% in 2014 compared with 83.5% and 84.3% in the CBS social survey respectively. Further information on ESS can be found in the survey's website: http://www.europeansocialsurvey.org/
